# Distal ureteral stone in an ileal conduit: A case treated by antegrade flexible ureteroscopic lithotripsy

**DOI:** 10.1002/ccr3.3180

**Published:** 2020-09-02

**Authors:** Hao‐Han Chang, Jhen‐Hao Jhan, Tsung‐Yi Huang

**Affiliations:** ^1^ Department of Urology Kaohsiung Medical University Hospital Kaohsiung Taiwan; ^2^ Kaohsiung Medical University Kaohsiung Taiwan; ^3^ Department of Urology Kaohsiung Municipal Siaogang Hospital Kaohsiung Taiwan

**Keywords:** flexible ureteroscopy, ureter stone, urinary diversion

## Abstract

Antegrade flexible URSL is a minimal invasive option for treating distal ureteral stones in patient after urinary diversion.

## Graphical abstract

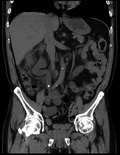

## 1 INTRODUCTION

Radical cystectomy with ileal conduit has been well established for treating muscle invasive bladder cancer. However, distal ureteral stone in an ileal conduit may pose a therapeutic challenge. We present a 68‐year‐old male who received radical cystectomy and ileal conduit 11 years ago developed a 0.7 cm right distal ureteral stone with right hydronephrosis and hydroureter. After resolving ureteral obstruction with percutaneous nephrostomy, we performed antegrade flexible ureteroscopic lithotripsy, effectively fragmented, and removed the stone. At a 1‐month follow‐up, there was no hydronephrosis and residual stone. This report attempted to demonstrate that antegrade flexible URSL is a reproducible, minimal invasive option for treating both the distal and proximal ureteral stones in patient after urinary diversion.

Ileal conduit has been considered one of the standard methods of urinary diversion following radical cystectomy. Urolithiasis, one of the common complications after ileal conduit, has a prevalence of approximately 9%‐15.3% based on a series study.^1^ However, treatment of ureteral stone following ileal conduit is quite challenging. Inserting the standard rigid ureteroscope (URS) into a reconstructed orifice is difficult, and a series study presented a too low success rate due to nonrecognition of neoureteral orifice.^2^ Therefore, we present a novel, reproducible method for treating ureteral stone after ileal conduit.

## 2 CASE REPORT

**FIGURE 1:**
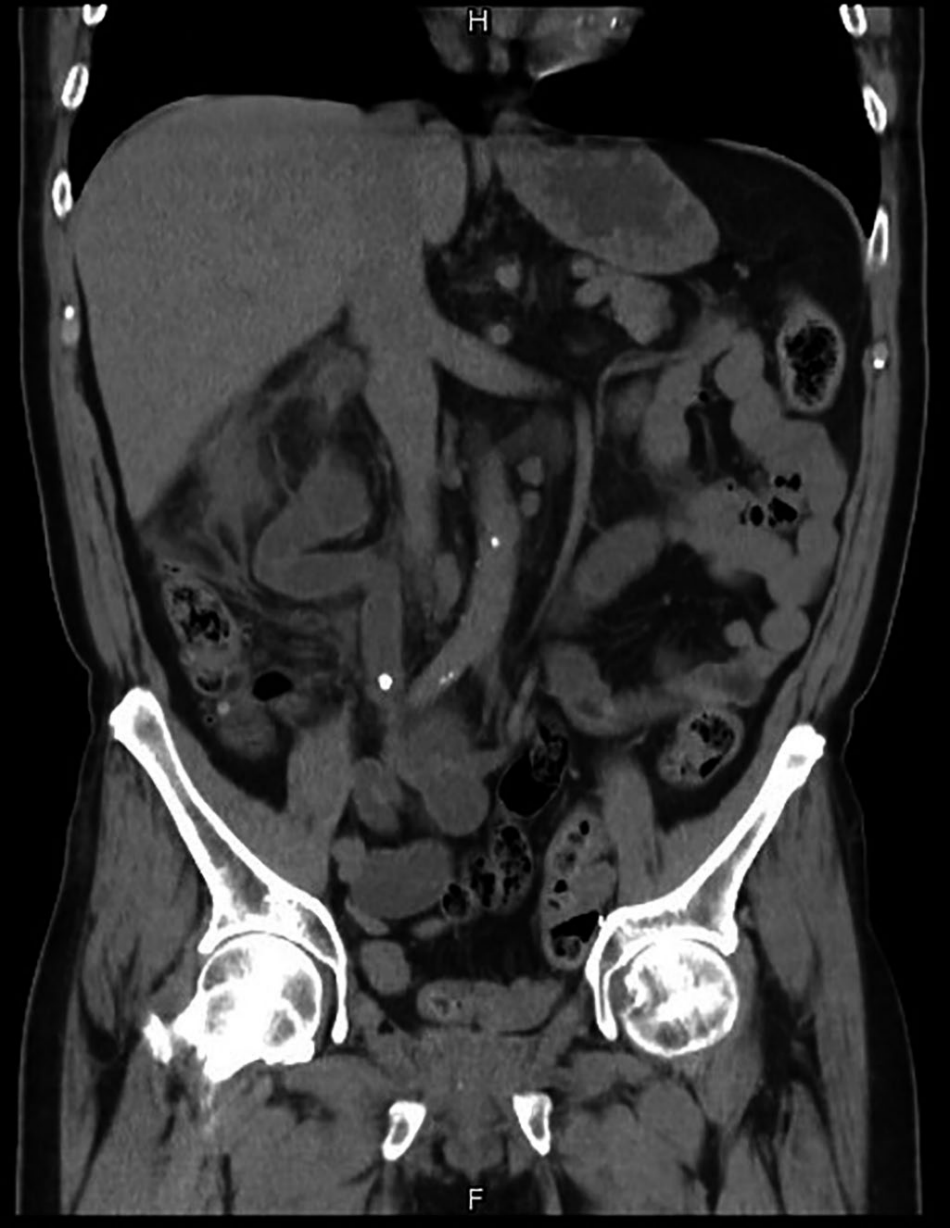
A 0.7‐cm distal ureteral stone near the ureteroileal anastomosis

A 68‐year‐old patient presented to our emergency department due to abdominal and right flank pain for 2 days. Tracing back the medical history revealed that the patient had received radical cystectomy and ileal conduit 11 years ago. Furthermore, he had developed a ureteral stone 3 years ago, which was resolved through a medical expulsive therapy. Physical examination revealed right flank knocking pain. Laboratory examinations revealed leucocytosis and an elevated creatine level. Initial ultrasound revealed right hydronephrosis. Noncontrast computed tomography (CT) revealed a 0.7‐cm impacted stone in the distal ureter, 2 cm away from ileal conduit orifice (Figure 1). Right hydronephrosis and fat stranding near the right kidney indicated nephritis of the right kidney. We inserted a 10 Fr tube in the middle calyx for percutaneous nephrostomy (PCN) under fluoroscopy for treating ureteral obstruction in the right kidney.

**FIGURE 2:**
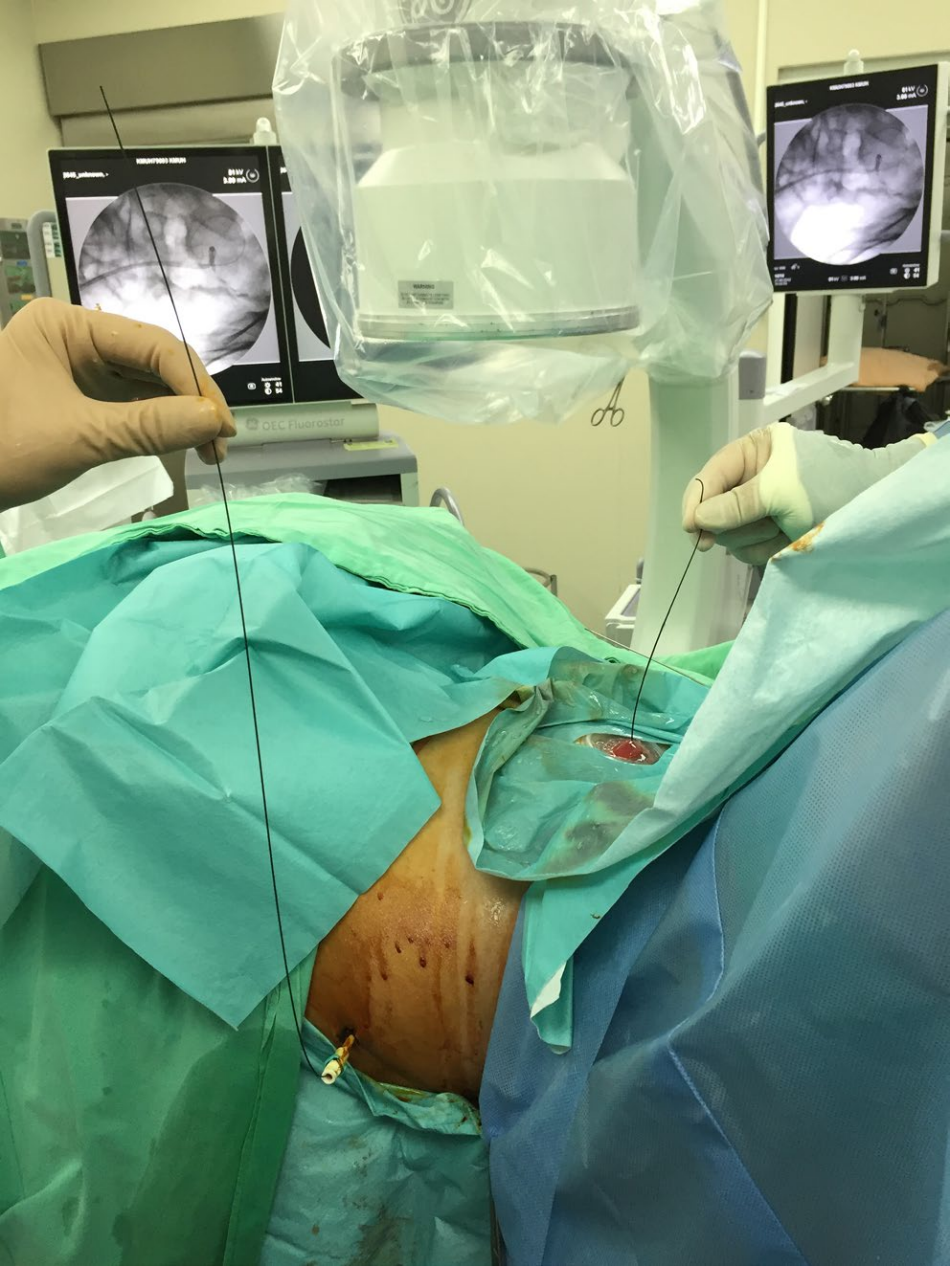
Communicating route set up for percutaneous nephrolithotomy through ileal conduit

**FIGURE 3:**
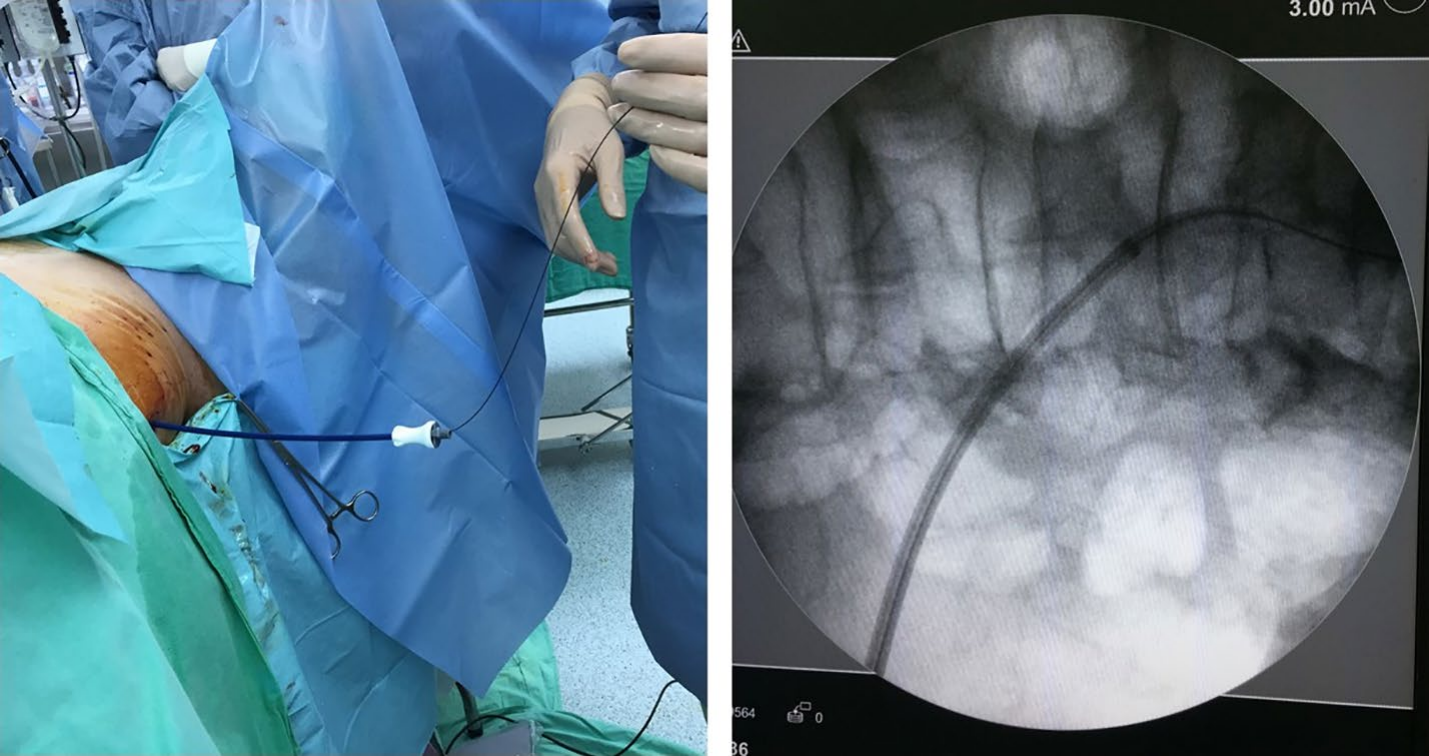
Dilation of the route to 12 F and insertion of an access sheath into ureter under fluoroscopy

**FIGURE 4:**
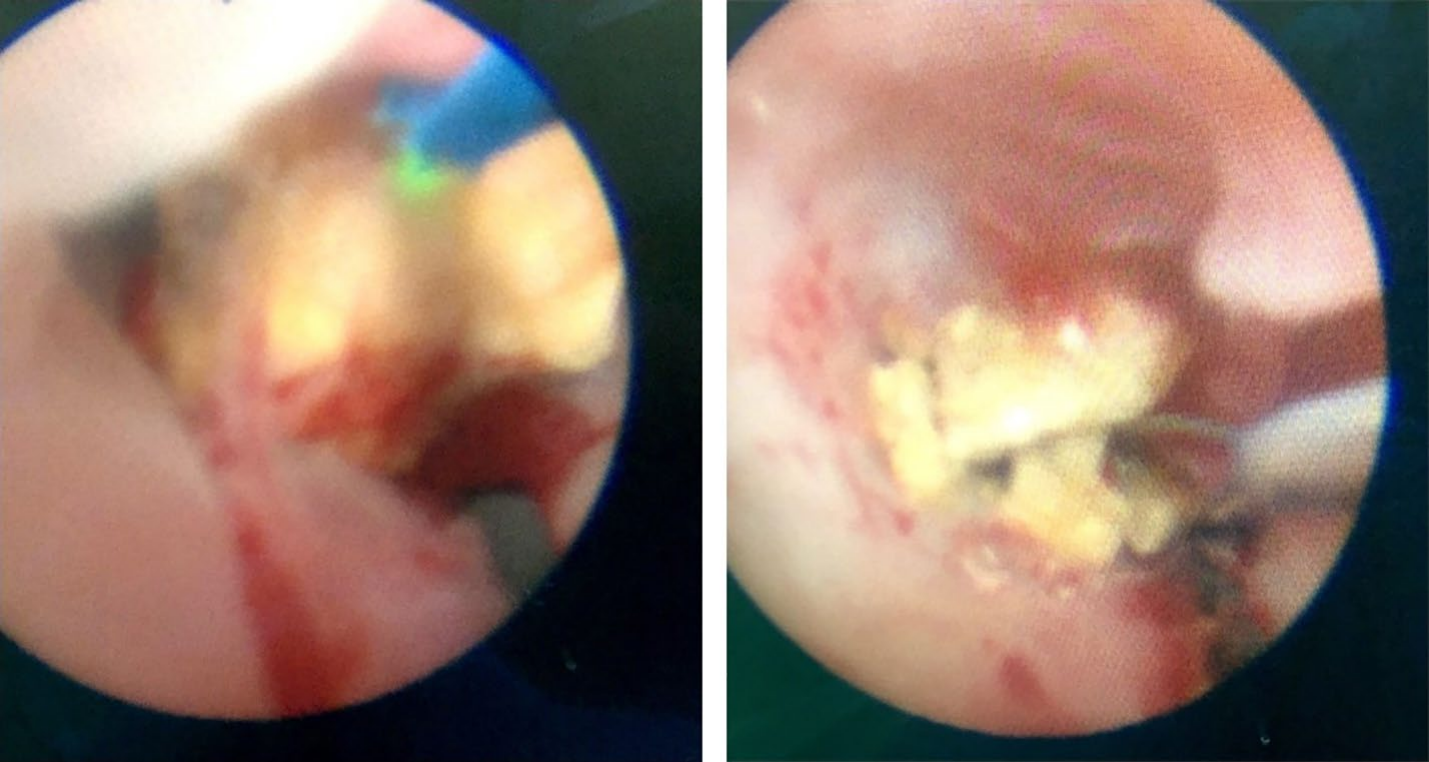
Locating the stone, fragmenting with a laser, and removing by using a stone basket

The operation was performed after 3 days of presentation. The patient was positioned in the Galdakao‐modified supine Valdivia position, similar to that in endoscopic combined intrarenal surgery, under general anesthesia. In this position, both ileal conduit and PCN can be performed simultaneously. A terumo guidewire was sent through the PCN tract to ileal conduit under fluoroscopy. Then, the other side of the guidewire was pulled out from ileal conduit by using a cystoscope and a pair of forceps, setting up a promising, communicating route (Figure 2). We attempted indwell rigid URS retrogradely into the ureter, but unfortunately it failed. Thus, the PCN wound was dilated to size 12 Fr, and an 11/13 Fr 36 cm access sheath (Boston scientific) was inserted into the middle ureter under flouroscopy (Figure 3). The guidewire was pulled out enough to leave the access sheath but remained inside the ureter from the ileal conduit part, thus maintaining enough space such that a 5.2/9.9 Fr flexible URS (fURS; Richard Wolf) could be introduced into the distal ureter through the access sheath antegradely. The stone was located in the distal ureter. We fragmented the stone by using a Holmium laser and removed by 1.5 Fr Nitinol stone basket (Cook) (Figure 4). A 6 Fr, 22 cm JJ tube was indwelled. A contrast medium was injected through the access sheath, and the access sheath was then pulled back to the renal pelvis, ensuring that no ureteral obstruction was present. After the access sheath was removed, the PCN wound was closed. The patient was uneventfully discharged. At follow‐up, kidney, ureter, and bladder (KUB) examination after 1 week revealed no residual stone. Afterward, stone analysis revealed a calcium oxalate stone.

## 3 DISCUSSION

Urolithiasis is a common complication in patients after urinary diversion. Several risk factors promoting stone formation have been reported, including bacterial colonization, urinary metabolic derangements, urinary stasis, reflux of mucus into the upper tract, and exposure to surgical material.^3^ The most common types of stones as revealed by stone analysis are struvite and calcium phosphate stones, with an incidence rate of 64% and 25%, respectively.^4^

In case of small asymptomatic urolithiasis, conservative treatment or medical expulsive therapy may always be the first option, whereas in case of symptomatic circumstance, several options have been reported in patient after ileal conduit, including PCNL, ureteroscopic lithotripsy (URSL), and SWL.^2^ Although improvement in the endoscopic technology and techniques has shifted the management of urolithiasis away from the traditional open surgery era, treating urolithiasis in ileal conduit is still a challenge. As recognizing the neoureteral orifice and traversing through ureteroenteric anastomosis may be extremely difficult,^4^ treating distal ureteral stone is more challenging than treating renal and upper ureteral calculi.

We attempted to develop a more promising, reproducible, and minimally invasive method through this case for treating a patient with distal as well as proximal ureteral stone after ileal conduit.

Two major difficulties existed for introducing fURS antegradely. The first was the angle formed by the calyx, wherein instruments for PCN were inserted, and the ureteropelvic junction (UPJ). The angle sharpness positively correlated with the difficulty level. To decrease this angle, we punctured into the middle calyx rather than the lower calyx. Performing the PCN procedure through the upper calyx can cause iatrogenic pneumothorax; however, if the procedure is performed with care, this complication can be avoided and the upper calyx can be a potential route for executing the operation. The second difficulty was the tortuous ureter caused by hydroureter. This situation could be solved by indwelling the access sheath rather than by merely introducing fURS. In addition, this maneuver could protect the fragile fURS from being damaged easily.

This operation presented several advantages after overcoming the aforementioned difficulties. First, it seemed to be a reproducible method that can be applied in different ways for urinary diversion, including ileal conduit and orthotopic neobladder. Second, the stone could be fragmented and removed by the surgeon, similar to traditional URSL and retrograde intrarenal surgery (RIRS), thus decreasing the learning curve of this operation.

According to our knowledge, although several articles have mentioned treating a patient with ureteral stone after ileal conduit through fURS antegradely, most of them did not report detailed operation steps and lack intraoperative images.

## 4 CONCLUSION

Urolithiasis in patient after urinary diversion is not completely understood. Treating these diseases is difficult, and the treatment should be customized by individual patients. Our surgical plan is modified based on the different characteristics of patients and location and size of their stone. This report attempted to demonstrate that antegrade flexible URSL is a reproducible, minimal invasive option for treating both the distal and proximal ureteral stones in patient after urinary diversion.

## CONFLICT OF INTEREST

None declared.
